# The impact of online teaching curricula on undergraduate basic surgical skills acquisition

**DOI:** 10.1016/j.sopen.2025.06.002

**Published:** 2025-06-06

**Authors:** Devansh Tandon, Ayush Gupta, Rhianna Patel, Anushka Shukla, Saran Singh Gill, Rhea Elise Patel, Keshav Krishnan, Bishoy Yassa, Shivansh Tandon, Amar Rai, Matt Boal, Nader Francis

**Affiliations:** a University College London Medical School, University College London, London, UK; b Imperial College London School of Medicine, Imperial College London, London, UK; c Leeds Medical School, University of Leeds, Leeds, UK; d Barts and The London School of Medicine and Dentistry, Queen Mary University of London, London, UK; e GKT School of Medicine, King's College London, London, UK; f Imperial College Healthcare NHS Trust, London, UK; g The Griffin Institute, Northwick Park and St Mark's Hospital, London, UK

## Abstract

**Introduction:**

Despite the growing use of online teaching in medical education, undergraduate surgical skills training remains predominantly face-to-face, with limited structured curricula and resources. Consequently, many students lack confidence performing basic procedures independently. While online programmes offer a potential alternative, comparative evidence is limited. This service evaluation assessed the effectiveness of online surgical skills teaching on student skill acquisition and confidence.

**Methods:**

Medical students who participated in five UK national surgical skills programmes between 2022 and 2024 were selected, having received either online or face-to-face instruction. Skill acquisition was measured using the Objective Structured Assessment of Technical Skills (OSATS) tool and confidence was measured pre- and post-training via a Likert scale. Non-parametric data were analysed using the Mann-Whitney *U* test, with significance set at *p* < 0.05.

**Results:**

Of 133 participants, 82 received online and 51 face-to-face training. Fifty-six percent were in their first or second year of study. No significant differences were found in continuous (*p* = 0.0652) or mattress suturing (*p* = 0.143), while interrupted suturing scores were significantly higher in the online group (*p* = 0.0143). Both modalities significantly improved confidence (*p* < 0.0001), with no significant difference between groups (*p* > 0.05).

**Conclusion:**

This study demonstrates that online surgical skills teaching is as effective as face-to-face methods, with both positively impacting skill acquisition and confidence. A hybrid approach, integrating online and face-to-face teaching, could optimise learning by combining the scalability of online instruction with essential practical experience, enhancing medical students' confidence and technical proficiency in surgical skills.

## Highlights

• No significant difference was observed in overall surgical skill acquisition between online and face-to-face teaching.
• Online teaching resulted in significantly higher proficiency in interrupted suturing compared to face-to-face sessions.
• These findings support a hybrid learning model, combining online and in-person methods.

## Introduction

In recent years, the integration of online teaching in undergraduate education has surged. A 2022 Medical Schools Council (MSC) survey, encompassing responses from 70 % of UK medical schools, reported that online teaching is now embedded within medical curricula [1]. Similar trends have been observed in other regions, including North America, Australia, and the Middle East, where digital learning platforms have been widely adopted to supplement traditional teaching methods [2,3]. Online teaching in undergraduate medical education is usually delivered either in real time or pre-recorded lectures or online courses completed within a fixed timeframe [4]. Online teaching enhances accessibility through flexible scheduling, enabling students to balance studies, clinical placements, and personal commitments while offering cost-effective solutions for both students and faculty [5]. This is particularly relevant for students in geographically-dispersed regions or low-resource settings, where face-to-face training opportunities are limited [6,7]. Together, these benefits create a more inclusive and equitable learning experience, supporting students from diverse backgrounds [[8], [9], [10], [11]].

The teaching of surgical skills is traditionally delivered face-to-face, both at the undergraduate level and throughout postgraduate specialty training [12]. In accordance with General Medical Council (GMC) guidelines as well as equivalent medical regulatory bodies worldwide such as the Accreditation Council for Graduate Medical Education (ACGME) in the US and the Australian Medical Council (AMC), medical students are typically expected to receive formal surgical skills training, centrally organised by medical schools. However, many students initially acquire these skills through surgical societies and external conferences rather than structured medical education curricula [13]. This reliance on extracurricular training has also been reported in studies from the US and Canada, where students often supplement limited formal training with workshops and independent learning [[14], [15], [16], [17]]. Despite the GMC's “Outcomes for Graduates” outlining a foundational surgical curriculum aimed at ensuring competency, undergraduate surgical education in the UK remains insufficiently comprehensive, with similar concerns raised globally [[13], [14], [15], [16], [17], [18]]. A survey of 705 medical students across the UK found that 86.5 % reported inadequate training in fundamental surgical techniques such as suturing, citing key concerns including limited curricular training and facilities, insufficient depth of suture instruction, and a strong desire for more hands-on experience [19]. Notably, while extracurricular training was identified as beneficial in enhancing procedural confidence, only 45 % of final-year students felt confident suturing unsupervised, likely due to insufficient practical exposure [19,20]. Comparable findings have emerged from European and Australasian studies, where students report variability in exposure to surgical skills training, impacting their confidence and preparedness for clinical practice [[21], [22], [23]].

In response to this deficit, numerous teaching programmes and surgical skills workshops have emerged, often organised by student societies and independent non-profit organisations, aiming to upskill medical students interested in surgery [[24], [25], [26]]. While many of these sessions continue to be delivered face-to-face, there is an increasing shift toward online formats, particularly through synchronous online seminars, which facilitate wider accessibility and larger audiences. Evidence suggests that online learning can provide an effective alternative when structured appropriately, particularly for procedural skill acquisition. Despite the paucity of direct comparisons between face-to-face and online surgical skills training, online modalities have been associated with positive objective outcomes [2,15,27,28]. However, these findings are often constrained by methodological limitations, often relying on subjective self-reported measures, while objective skill assessments remain scarce, making it challenging to draw definitive conclusions about their comparative effectiveness [16].

As such, this study aimed to address this gap in the literature by analysing data from numerous basic surgical skills programmes using the Objective Structured Assessment of Technical Skill (OSATS) framework, a validated and widely recognised tool for assessing technical skill proficiency and progression to therefore assess the efficacy of online basic surgical skills teaching on a broader, internationally-relevant scale [29]. By employing objective and standardised evaluation criteria, this service evaluation aims to provide robust and evidence-based insights into the comparative effectiveness of online versus face-to-face surgical training.

## Methods

Five basic surgical skills teaching programmes were evaluated for methodological consistency, ensuring a uniform approach to skill acquisition and assessment. Selection of these programme was carried out using shortlisting criteria which included a shared focus on fundamental skills, a structured session format (see supplementary materials), the use of a standardised OSATS tool (supplementary materials 1), and comparable feedback forms for consistent participant evaluation (supplementary materials 2). Table 1 defines the characteristics of each included teaching programme.

**Table 1:** Teaching programmes included in evaluation.

Organiser	Participants	N	Session	Modality	Skills Assessed
British Indian Surgical Association (BISA)	Medical students attending annual programmes (2022–2024)	84	90-minute sessions covering 5–6 basic surgical skills	Online (synchronous, live video conferencing)	Suturing, knot tying, wound closure, instrument handling
Leeds Cutting Edge Surgical Society (LCESS)	Medical students attending 2023 programme	30	90-minute sessions covering 5 basic surgical skills	Face-to-face	Suturing, knot tying, wound closure, laparoscopic skills
The Griffin Institute, St Marks Hospital	UCL medical students intercalating in Surgical Sciences (2022)	24	Two 3-hour sessions over 2 weeks covering basic and advanced surgical skills	Face-to-face	Basic and advanced suturing, knot tying, tissue handling, electrosurgery

### Outcomes

Across all five teaching programmes at the end of each session, assessment of skill acquisition was measured using a validated task-specific OSATS tool for each participant. Tutors scored participants' OSATS scores out of 21 points for each surgical skill, with 16 points available for technique and 5 points as part of a global rating scale. A 5-point Likert scale determining participant confidence pre and post teaching was measured through a feedback form. A score of 0 was equivalent to ‘strongly disagree’ and a score of 5 was equivalent to ‘strongly agree’. OSATS was selected as the primary outcome due to its consistent use across programmes, while changes in participant confidence pre- and post-session served as a secondary measure. Only participants with recorded OSATS scores and completed post-session feedback were included.

### Data synthesis

Respective organisations and societies were contacted with a request to access the data from each teaching programme and permission sought to use the data. Anonymous demographic data was presented as descriptive statistics in Table 2. OSATS scores for each suturing technique and pre- and post-session confidence scores were then pooled into online and face-to-face teaching groups for analysis. Subcuticular suturing and knot tying were excluded from analysis due to insufficient data in face-to-face sessions.

**Table 2:** Table of demographics of online and face-to-face participants.

Demographics	Number of Online Participants (%) *N* = 82	Number of Face-to-Face Participants (%) *N* = 51	p
Stage of Medical School			
Year 1 medical student	26 (31.7 %)	12 (23.5 %)	<0.001
Year 2 medical student	28 (34.1 %)	8 (15.7 %)	
Year 3 medical student	19 (23.2 %)	25 (49.0 %)	
Year 4 medical student	4 (4.9 %)	2 (4.0 %)	
Year 5 medical student	0 (0.0 %)	4 (7.8 %)	
Intercalated BSc	5 (6.1 %)	0 (0.0 %)	
University of Study			
University of Aberdeen	2 (2.4 %)	0 (0.0 %)	<0.001
Bart's and the London School of Medicine	1 (1.2 %)	0 (0.0 %)	
University of Birmingham	1 (1.2 %)	0 (0.0 %)	
Bristol University	2 (2.4 %)	0 (0.0 %)	
University of Dundee	1 (1.2 %)	0 (0.0 %)	
Hull York Medical School	1 (1.2 %)	0 (0.0 %)	
Imperial College London	20 (24.4 %)	0 (0.0 %)	
Keele University	1 (1.2 %)	0 (0.0 %)	
King's College London	17 (20.7 %)	0 (0.0 %)	
University of Lancashire	4 (4.9 %)	0 (0.0 %)	
University of Leeds	2 (0.5 %)	0 (0.0 %)	
University of Liverpool	1 (1.2 %)	27 (52.9 %)	
Queen's University Belfast	1 (1.2 %)	0 (0.0 %)	
University of Manchester	1 (1.2 %)	0 (0.0 %)	
University of Nottingham	6 (7.3 %)	0 (0.0 %)	
St George's University of London	3 (3.7 %)	0 (0.0 %)	
Southampton Medical School	1 (1.2 %)	0 (0.0 %)	
University of Sheffield	2 (2.4 %)	0 (0.0 %)	
University College London	15 (18.3 %)	24 (47.1 %)	
Sex			
Male	33 (40.2 %)	23 (45.1 %)	0.711
Female	49 (59.8 %)	28 (54.9 %)	
Hand Dominance			
Right-handed	51 (62.2 %)	25 (49.0 %)	0.786
Left-handed	7 (8.5 %)	2 (3.9 %)	
Unreported	24 (29.3 %)	24 (47.1 %)	
Ethnicity			
African	1 (1.2 %)	2 (3.9 %)	0.001
South Asian	47 (57.3 %)	10 (19.6 %)	
East Asian	1 (1.2 %)	0 (0.0 %)	
Middle Eastern	1 (1.2 %)	2 (3.9 %)	
White	8 (9.8 %)	13 (25.5 %)	
Unreported	24 (29.3 %)	24 (47.1 %)	

*p*-value for university distribution is from a chi-squared test; interpret with caution due to multiple zero counts violating test assumptions.

### Statistical methods

Statistical analysis and figure generation was performed using Microsoft® Excel Version 16.93.1 (Microsoft Corporation, Redmond, WA, USA). Normality was assessed via the Shapiro-Wilk test. As the data was non-parametric, pre- and post-session differences were analysed using the Mann-Whitney test. Statistical significance was set at *p* < 0.05.

### Post-hoc power calculation

A post-hoc power analysis was performed to assess the statistical sensitivity of the comparisons between online and face-to-face OSATS scores across suturing techniques. Cohen's d was estimated using the difference in medians as a proxy for the mean, and interquartile range (IQR) divided by 1.35 as an approximation for standard deviation [30]. Effect sizes were then converted to rank-biserial correlation coefficients to align with the non-parametric Mann-Whitney *U* test used for group comparisons. Statistical power was calculated using standard formulas for independent group comparisons, with α set at 0.05 and actual group sizes used for each suturing technique.

## Results

### Baseline characteristics

A total of 133 participants (96 % of those registered) attended both online and face-to-face surgical skills sessions, with 82 attending online and 51 attending face-to-face. The majority of participants were in Year 1 or Year 2 (31.7 % and 34.1 % respectively), representing 19 medical schools across the UK, though only University College London and University of Leeds had participants in the face-to-face sessions. There were statistically significant differences in stage of medical school (*p* < 0.001), ethnicity (*p* = 0.0013), and university affiliation (p < 0.001) between groups, while sex (*p* = 0.711) and hand dominance (*p* = 0.786) were comparable (Table 2).

### OSATS

**Fig. 1:**
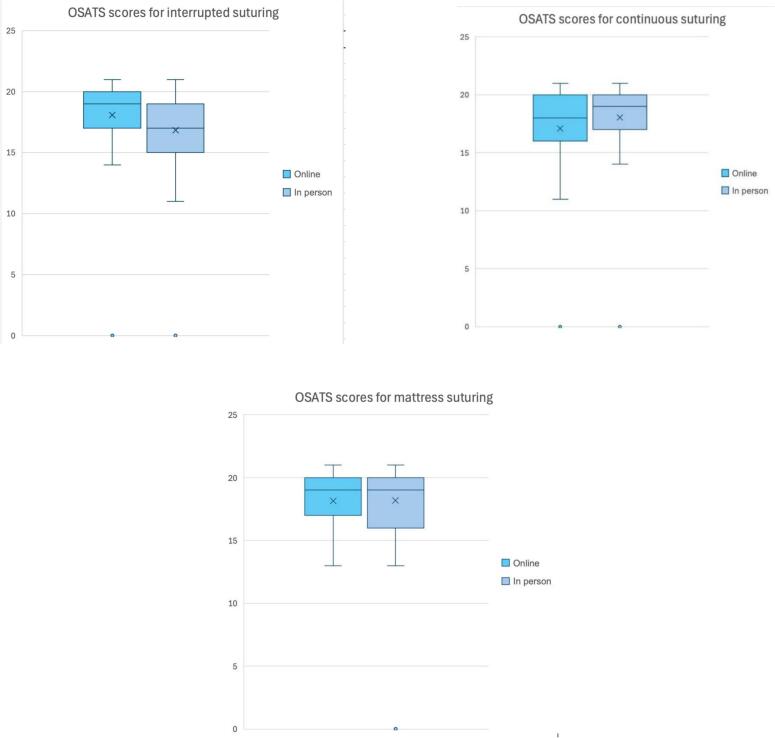
Box and whisker plots representing and comparing OSATS Scores between online and face-to-face attendees. Each graph represents a different suture type – continuous, interrupted and mattress.

A statistically significant difference was observed in interrupted suturing skill scores for the online group compared to face-to-face teaching (19[3] vs. 17[3.5], *p* = 0.0143). However, no significant differences were noted for continuous suturing (17.5[3.25] vs 19[3], *p* = 0.0652) or mattress suturing (19[3] vs 19[3.5], *p* = 0.525) (Table 3) (Fig. 1).

**Table 3:** Table of median OSATS scores for each suture type for both online and face-to-face course. The data is non-parametric and unpaired therefore the Mann-Whitney-U test was used to compare the medians. *Statistically significant difference between medians at a 0.05 significance level.

Suturing type	Online OSATS score (median (IQR))		Face-to-face OSATS Score (median (IQR))		p-value
Continuous	*n* = 60	17.5 (3.25)	*n* = 44	19.0 (3)	0.0652
Interrupted	*n* = 61	19 (3)	n = 51	17 (3.5)	0.0143^⁎^
Mattress	*n* = 55	19 (3)	*n* = 29	19 (3.5)	0.525

### Participant confidence

Both online and face-to-face teaching resulted in a statistically significant increase in participant confidence from pre- to post-session (*p* < 0.0001). However, when directly comparing confidence gains between modalities, no significant difference was found (*p* > 0.05) (Table 4, Table 5).

**Table 4:** Table demonstrating pre and post session Likert scores for both face-to-face and online attendees and subsequent statistical analysis within each group. Statistical analysis utilised the Mann-Whitney-U test to see if there was a significant difference between the medians. *Statistically significant difference between medians at a 0.05 significance level.

Session type	Question	Pre-session (median (IQR)) (*n* *=* *29*)	Post-session (median (IQR)) (*n* *=* *28*)	p-value
Face-to-face	Rate your confidence in basic surgical skills (knot tying, interrupted/continuous/mattress sutures)	1 (1)	4 (2)	<0.0001^⁎^
	Rate your confidence in your knowledge of surgical instruments (relevant to basic surgical skills)	2 (1)	4 (1)	<0.0001^⁎^
Online	Rate your confidence in basic surgical skills (knot tying, interrupted/continuous/mattress sutures)	1 (1)	4 (1)	<0.0001^⁎^
	Rate your confidence in your knowledge of surgical instruments (relevant to basic surgical skills)	2 (1.25)	4 (0)	<0.0001^⁎^

**Table 5:** Table illustrating the change in confidence levels pre and post session, measured using Likert scales and statistically compared for face-to-face and online teaching. Statistical analysis utilised the Mann-Whitney-U test.

Question	Face-to-face: median of change in confidence between pre- and post-feedback (*n* *=* *29*)	Online: median of change in confidence between pre- and post-feedback (*n* *=* *28*)	p-value
Rate your confidence in basic surgical skills (knot tying, interrupted/continuous/mattress sutures)	2	3	0.1156
Rate your confidence in your knowledge of surgical instruments (relevant to basic surgical skills)	3	2.5	0.2592

### Post-hoc power calculation

Post-hoc power analysis indicated that the study was well-powered to detect the observed difference in interrupted suturing proficiency, with a large effect size (d = 0.834, r₍rb₎ = 0.385) and an estimated power exceeding 90 %. For continuous suturing, the moderate effect size (d = −0.643, r₍rb₎ = −0.306) was associated with lower statistical power (approximately 50 %), suggesting a heightened risk of type II error. Mattress suturing, with no observable effect (d < 0.001, r₍rb₎ < 0.001), had negligible power to detect a difference, consistent with the lack of group separation.

## Discussion

This study comprehensively evaluated the basic surgical skills acquisition of UK undergraduate medical students following delivery of online and face-to-face teaching curricula. As per curriculum validation, such as Kirkpatrick's model, this study evaluated participant reaction and behaviour through feedback, as well as objectively assessing skill acquisition [31,32]. Students in the online group demonstrated a similar level of skill acquisition as the face-to-face group in continuous and mattress suture techniques. This suggests that the online teaching modality may be a viable method of delivering surgical training, particularly in regions where access to face-to-face training is limited. Students performing interrupted sutures in the online group outperformed those in the face-to-face group, highlighting the potential of online teaching as an adjunct to traditional face-to-face teaching.

The finding that online training was associated with significantly higher OSATS scores for interrupted suturing, compared to face-to-face instruction, warrants further investigation into the pedagogical and contextual factors that may have contributed to this outcome. Online formats often enable asynchronous learning, allowing students to practise at their own pace and revisit instructional videos, which can reinforce procedural memory and motor skills acquisition [33,34]. The home-based environment may also reduce performance anxiety typically associated with peer or tutor observation, helping learners to focus more effectively on technique. Moreover, online participants may have had greater autonomy over their schedules and more opportunities to engage in repeated, self-directed practice and rewatching of the material, using consistent audiovisual materials. These features, aligned with principles of adult learning theory, may have disproportionately benefited the online group in this task, potentially skewing the results [35,36]. Additionally, the skew toward high OSATS scores in both groups, with the lowest median (17/21) observed in the face-to-face cohort for interrupted suturing, suggests that students across both modalities performed well overall. This may partly reflect the greater opportunity for practice afforded by online learning. According to Hiemstra et al., a score of 81 % constitutes good surgical performance in the absence of formal benchmarks [37]. Notably, both online and face-to-face formats led to significant improvements in participant confidence (*p* < 0.0001), reinforcing the value of both modalities. Together, these findings highlight the effectiveness of structured surgical skills training in enhancing both competence and confidence at the undergraduate level, while also underscoring the need for further research into how delivery format influences learning outcomes.

A recent systematic review by Glossop et al., assessing 19 studies, found a ‘concerning lack of surgical skills teaching’ at undergraduate level and the resulting discrepancy between the GMC's guidance for medical education and current UK medical curriculum [38]. Similar trends have been reported in the US, Australia, and Europe, where medical graduates often feel underprepared for core surgical skills due to inconsistent curriculum integration [3,[14], [15], [16], [17]]. Any undergraduate surgical skills teaching provided is often non-technical, brief and inconsistent, as well as taking place later in the clinical years of medical school [13,39]. This deficiency in surgical skills training is particularly concerning given the current workforce challenges not just within the NHS but also in other healthcare systems globally. The shortage of surgical trainees, coupled with increasing service demands, places greater reliance on junior doctors to perform basic surgical tasks, often with limited formal training. In regions facing surgical workforce shortages, such as sub-Saharan Africa and parts of Asia, early surgical training in medical school is crucial for ensuring competency in essential procedures [2,6,7]. A lack of early exposure and competency in basic surgical techniques not only impacts patient safety and procedural efficiency but also contributes to workplace stress and burnout among junior doctors, who may feel underprepared for essential surgical responsibilities [40].

Furthermore, the NHS Long Term Workforce Plan highlights the need for early skills development to improve workforce retention and efficiency [41]. Equipping medical students with foundational surgical competencies could facilitate a smoother transition into clinical roles, reducing the burden on postgraduate training and enhancing surgical career preparedness. Considering increasing attrition rates among surgical trainees and the growing emphasis on multi-disciplinary team-based care, structured and accessible surgical skills teaching is essential to future-proof the NHS workforce and ensure high standards of patient care. Given the constraints on face-to-face teaching resources, online surgical skills training presents a scalable and cost-effective alternative, capable of reaching a broader cohort of students. Integrating high-quality, standardised surgical education at the undergraduate level could help bridge the gap between GMC guidelines and real-world clinical practice, ensuring that future doctors enter the workforce with greater confidence and competency in core surgical skills.

Moreover, there is significant variation in the role that surgical skills teaching plays within the undergraduate curricula across different medical schools [13,38]. Internationally, a more unified approach to integrating structured surgical skills training could help standardise competency levels among medical graduates. This could be addressed by integrating high-quality surgical skills teaching into the medical education curriculum. Iyer P et al. develop a six session surgical teaching series, held online and face-to-face, to teach skills such as suturing and theoretical knowledge, with both modalities showing significant improvements (*p* < 0.001) in understanding on a Likert scale [28]. While comparisons between online and face-to-face teaching were not performed as part of the study, this consolidates the message in our study: both modalities are viable methods of teaching surgical skills. Despite participants finding face-to-face sessions more engaging, outcomes in Iyer P et al.'s study did not impact levels of attainment [28]. More research is needed directly comparing effectiveness of online vs. face-to-face teaching but recent research is assuredly providing increasing evidence to support at least the consideration of an online adjunct [[42], [43], [44], [45], [46], [47]]. In the post-COVID-19 era, our study provides clear evidence that online teaching of basic surgical skills could serve as an adjunct to the medical curriculum, a concept supported by studies such as Mao BP et al. [3,10,[48], [49], [50], [51], [52]]. Their systematic review and meta-analysis of 11 studies, involving 715 participants, found no significant differences between video-based learning and traditional methods in terms of surgical skill proficiency, confidence, or satisfaction rates. Consequently, Mao BP et al. advocate for the integration of online education alongside traditional teaching methods to enhance surgical training [3,46]. In regions with limited access to face-to-face training, Cao et al.'s meta-analysis has shown that hybrid learning models combining online and face-to-face teaching yield superior outcomes compared to either method alone, suggesting that integrating both approaches could enhance surgical training worldwide [52].

Therefore, to adequately prepare students for an era of telemedicine and digital health, a hybrid model where online teaching is provided as an adjunct to face-to-face teaching could be considered. This approach has been explored in various global medical education systems, with promising outcomes in blended learning methodologies. This concept of ‘blended learning’ is particularly active in recent research, often yielding positive effects on knowledge outcomes from blended learning methods [48]. Our research aligns with literature, suggesting that the multimodal teaching of basic surgical skills could be the most effective strategy in addressing the dearth of surgical teaching within the medical curriculum [26,42,[53], [54], [55]]. Yet, in order to implement this on a larger scale, several logistical factors must be accounted for, including ensuring the availability of an adequate number of tutors. A favourable teacher to student ratio, for instance, was utilised across all teaching programmes included in our study, generally varying from 1:6 to 1:4. The current literature suggests that an ideal ratio typically falls within this range, ensuring adequate supervision, personalised feedback, and effective skill acquisition in surgical education [56]. Although this boosts the likelihood of students acquiring the skills, maintaining a high teacher to student ratio could become difficult, should such a programme be integrated into the national curriculum. To further maximise student learning and maintain a high standard of teaching quality, expert surgeons with prior surgical and teaching experience were used. Similarly, this could be challenging to upscale nationally.

While this study represents the one of the first comparisons of online and face-to-face surgical skills training in undergraduate medical education to date, several limitations must be acknowledged. As a non-randomised service evaluation with a limited sample size, the study was not sufficiently powered to detect modest differences in all outcomes, particularly for continuous and mattress suturing, which reduces the generalisability of these findings. Differences in baseline characteristics between the online and face-to-face groups, including stage of medical school, ethnicity, and university affiliation, may have introduced confounding effects. No statistical adjustment or propensity score matching was undertaken, as the primary objective was to explore real-world outcomes across diverse programme settings rather than to establish causality. As such, findings should be interpreted with caution. Variability in participants' prior exposure to surgical skills, along with differences in training duration, feedback provision, and tutor expertise, may also have influenced both objective performance and subjective confidence outcomes. Furthermore, OSATS scores were recorded by different assessors in most sessions, with between 3 and 10 trainers present per session across multiple centres. Due to logistical constraints, no inter-rater reliability training or standardisation was implemented, which may have introduced scoring variability and reduced consistency in performance evaluation. This lack of standardised calibration may have introduced scoring variability, and future studies should aim to incorporate assessor training and report inter-rater reliability metrics, such as intraclass correlation coefficients, to enhance methodological robustness.

## Conclusion

This study demonstrates that online surgical skills teaching is as effective as traditional face-to-face methods, with both modalities positively impacting skill acquisition and significantly improving participant confidence. Notably, students learning online performed equally well in continuous and mattress suturing and outperformed their face-to-face counterparts in interrupted suturing, indicating that online teaching does not impede practical skill development. Furthermore, the accessibility and flexibility of online training addresses gaps in surgical education, particularly amid increasing NHS workforce pressures. A blended approach, integrating online and face-to-face teaching, could optimise learning outcomes by leveraging the scalability of online instruction while preserving essential hands-on experience. This model would enhance both the perceived confidence and technical proficiency of medical students, ensuring a more comprehensive and accessible surgical education.

## CRediT authorship contribution statement

**Devansh Tandon:** Conceptualization, Investigation, Methodology, Project administration, Writing – original draft. **Ayush Gupta:** Investigation, Project administration, Writing – original draft. **Rhianna Patel:** Data curation, Investigation, Project administration, Writing – original draft. **Anushka Shukla:** Data curation, Investigation, Writing – original draft. **Saran Singh Gill:** Conceptualization, Investigation, Project administration, Writing – review & editing. **Rhea Elise Patel:** Investigation. **Keshav Krishnan:** Investigation. **Bishoy Yassa:** Writing – review & editing. **Shivansh Tandon:** Investigation. **Amar Rai:** Conceptualization, Writing – review & editing. **Matt Boal:** Supervision, Writing – review & editing. **Nader Francis:** Conceptualization, Supervision, Writing – review & editing.

## Ethical approval

In line with guidance from Kings College London Research Ethics Office, this service evaluation did not require ethical approval as all data evaluated was pre-existing and anonymous at the point of access. No primary data collection was carried out as part of this work.

## Funding

The authors did not receive any support or funding from any organisation for this work.

## Declaration of competing interest

The authors report no conflict of interest in this work.

## Data Availability

Available from host organisations on reasonable request.

## Appendix A Supplementary data

Supplementary material Image 1
