# Supplementary material for: The impact of online teaching curricula on undergraduate basic surgical skills acquisition
Source: Surg Open Sci. 2025 Jun 6;27:8–14. doi: 10.1016/j.sopen.2025.06.002 (PMC12213259; doi:10.1016/j.sopen.2025.06.002)
Supplement: Supplementary file 1 — Supplementary material [file mmc1.docx]

**Supplementary materials 1: Further details of teaching programmes**

**BISA Basic Surgical Skills Teaching Programme(s):**

*Participant selection*

Teaching was open to all undergraduate medical students across the UK and students were selected on a first-come first-serve basis through a sign-up form advertised nationally. This sign-up form collected participant demographic details. Note: identifiable participant data was destroyed after each teaching programme, thereby anonymising the demographic data.

*Session structure*

All sessions within the programme for a particular year were delivered in consecutive weeks. All sessions were delivered by doctors, ranging from specialty trainees to professors in surgery. A minimum of 4 doctors were present at each session, ensuring individualised teaching and feedback, thereby upholding quality of teaching and assessment. At the start of each session, students and doctors were divided into breakout rooms with an average ratio of 1 doctor per 5 students. First, the doctor explained the use and importance of the skill being taught. Next, the doctor demonstrated how to perform the skill. The student then practiced the skill under supervision and received targeted feedback. Finally, the doctor carried out an objective assessment of skill acquisition via a validated task-specific OSATS tool scoring at the end of the session.

*Participant and programme award*

Students were awarded a certificate from BISA at the end of the programme, with attendance at all sessions mandatory.The 2022 basic surgical skills teaching programme organised by BISA was awarded CPD points by The Royal College of Surgeons of England. The 2023 and 2024 programmes were awarded CPD hour by The Royal College of Surgeons of Edinburgh.

**Leeds Cutting Edge Surgical Society Basic Surgical Skills Teaching Programme:**

*Participant selection*

Teaching was open to all undergraduates at Leeds Medical School who were selected on a first-come first-serve basis through a sign-up form advertised locally. This sign-up form collected participant demographic details. Note: identifiable participant data destroyed after each teaching programme, thereby anonymising the demographic data.

*Session structure*

6 sessions were delivered in consecutive weeks, covering the 5 skills with 1 summary session. All sessions were delivered by doctors, ranging from specialty trainees to professors in surgery. A minimum of 6 doctors were present at each session, ensuring individualised teaching and feedback, thereby upholding quality of teaching and assessment. At the start of each session, students and doctors were divided into groups with an average ratio of 1 doctor per 5 students. First, the doctor explained the use and importance of the skill being taught. Next, the doctor demonstrated how to perform the skill. The student then practiced the skill under supervision and received targeted feedback. Finally, the doctor carried out an objective assessment of skill acquisition via OSATS scoring at the end of the session.

*Participant award*

Students were awarded a certificate from Cutting Edge Surgical Society at the end of the programme, with attendance at all sessions mandatory.

**The Griffin Institute Basic Surgical Skills Teaching Programme:**

*Participant selection*

Teaching was specifically delivered to students who were pre-enrolled for this programme as part of UCL’s intercalated Surgical Sciences degree. Note: identifiable participant data destroyed after the surgical skills teaching programme, thereby anonymising the demographic data.

*Session structure*

All sessions were delivered by doctors, ranging from specialty trainees to professors in surgery. A minimum of 3 doctors were present at each session, ensuring individualised teaching and feedback, thereby upholding quality of teaching and assessment. First, the doctor explained the use and importance of the skill being taught. Next, the doctor demonstrated how to perform the skill. The student then practiced the skill under supervision and received targeted feedback. Finally, the doctor carried out an objective assessment of skill acquisition via OSATS scoring at the end of the session.

**Supplementary materials 2: OSATS scoring tool**

**Objective Structured Assessment of Technical Skills (OSATS): Suturing**

Candidate name:                                                       Self-assessment: yes/no (select appropriate):                                                                  Tutor:                                                            Date:

| **Checklist** | **Yes** | **No** |
| --- | --- | --- |
| Selects appropriate instruments |  |  |
| Needle loaded ½ to 2/3 from tip |  |  |
| Bite depth and distance from wound edge appropriate 0.5cm-1cmm |  |  |
| Needle enters tissue perpendicular (90 degree) |  |  |
| Single attempt taking bites |  |  |
| Forceps used to hold skin |  |  |
| Supinates wrist |  |  |
| Approximates wound edges (appropriate eversion) |  |  |
| Secures square knot with hand or instrument tie - surgeons knot first throw |  |  |
| Appropriate number of throws i.e. 3-4 braided, 6-7 monofilament |  |  |
| Sutures placed appropriate distance apart/equal bites each side |  |  |
| Cuts suture tail correct length |  |  |
| Avoids handling needle with fingers |  |  |
| Avoids torqueing skin i.e. sutures placed incorrectly |  |  |
| Avoids grasping needle tip |  |  |
| Avoids multiple forceps grasps on tissue/damaging tissue or foam pad |  |  |
| Score                          /16 | | |

Task:

**Global rating scale (Please mark/highlight)       /5**

**1**- Poor technique, poor manual dexterity/instrument handling and unacceptable knot/closure

**2**- In between 1 and 3

**3** - Moderately good technique, moderate manual dexterity/instrument handling, acceptable knot

**4** - In between 3 and 5

**5-** Excellent Technique, excellent manual dexterity, and excellent knot

**Supplementary materials 3: Pre and post course feedback forms**


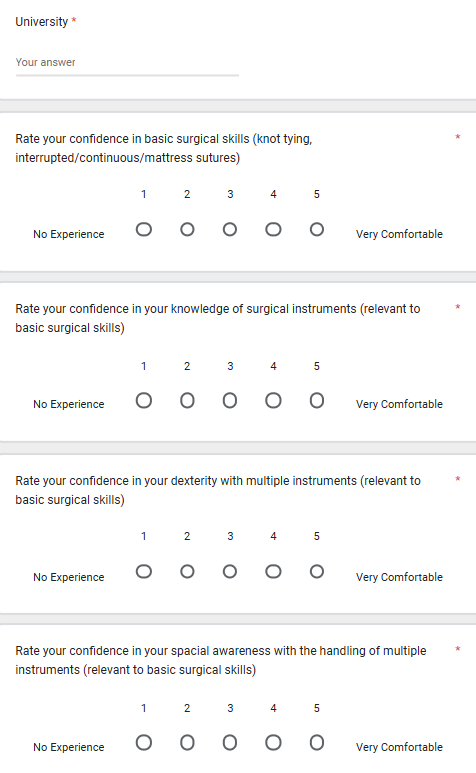

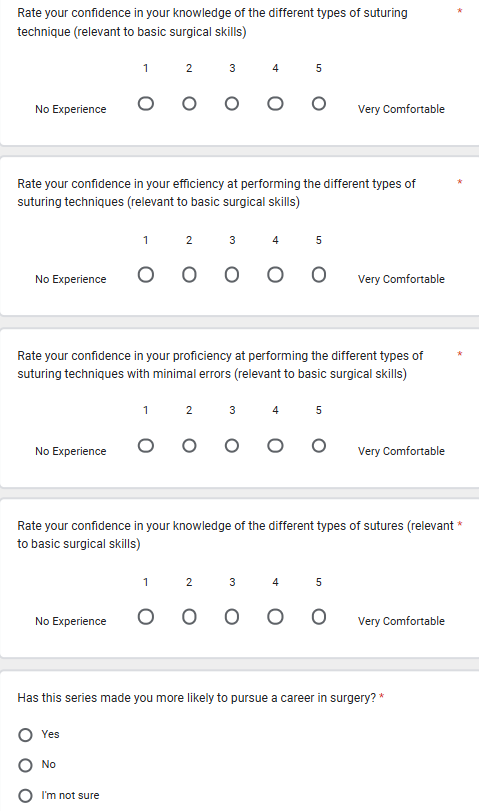

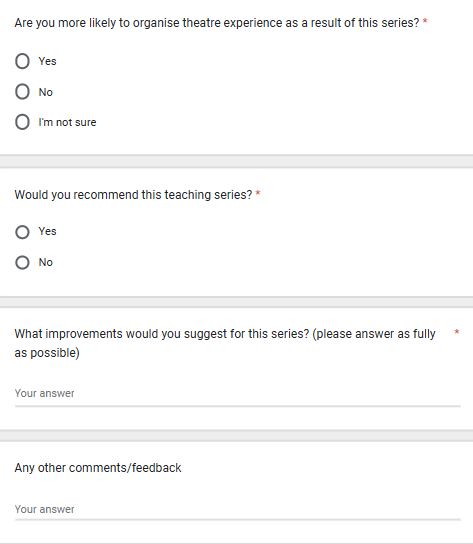

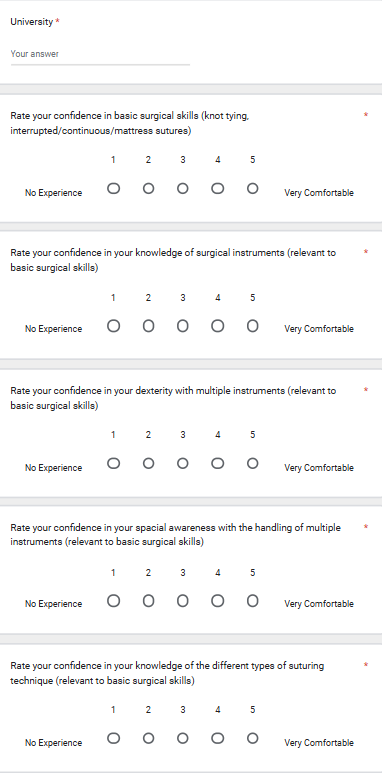

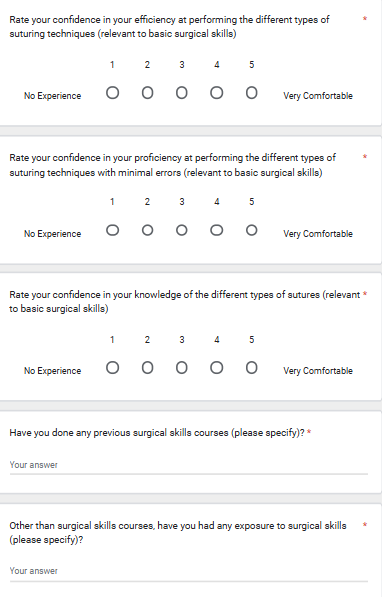


**Supplementary materials 4: Post-hoc Power Calculations**

| **Suturing Type** | **Cohen's d** | **Rank-Biserial r (r_rb)** | **Interpretation of Effect Size** | **Estimated Power (%)** | **Power Interpretation** |
| --- | --- | --- | --- | --- | --- |
| **Interrupted** | 0.834 | 0.385 | Large | 93 | Adequately powered to detect observed effect |
| **Continuous** | -0.643 | -0.306 | Moderate | 51 | Underpowered; possible type II error |
| **Mattress** | <0.001 | <0.001 | None | 5 | Negligible power due to no effect |
